# Double-edged sword: effects of human sperm reactive oxygen species on embryo development in IVF cycles

**DOI:** 10.1186/s12958-022-01053-7

**Published:** 2023-01-04

**Authors:** Jin Liu, Keheng Zhu, Shiyan Xu, Wenjiao Tu, Xiaotan Lin, Youpeng Su, Rong Huang, Yuao Deng, Yu Liu

**Affiliations:** 1 Department of Reproductive Medicine Center, Shenzhen People’s Hospital (The Second Clinical Medical College, Jinan University; The First Affiliated Hospital, Southern University of Science and Technology), Shenzhen, 518020 Guangdong China; 2 Department of Epidemiology and Biostatistics, School of Public Health, Tongji Medical College, Huazhong University of Science and Technology, Wuhan, 430030 China

**Keywords:** Embryo quality, Hydrogen peroxide, In vitro fertilization, Ovulation disorder, Sperm mitochondrial superoxide anion

## Abstract

**Background:**

The exact role of sperm reactive oxygen species (ROS) in early embryo development has yet to be fully identified, and most of existing research did not differentiate female infertility factors, ignoring the importance of oocyte quality in embryo development and the large differences in oocyte quality in women with infertility of different etiologies. And there has been no relevant report on whether different types of sperm ROS have distinct effects on embryo development. This study aimed to study the impact of selected sperm ROS, namely, sperm mitochondrial ROS (mROS) and hydrogen peroxide, on human embryo development after conventional in vitro fertilization (IVF) cycles in patients with normo-ovulatory infertility vs. anovulatory infertility.

**Methods:**

This was a prospective investigation including 393 couples underwent IVF cycles, among whom 90 patients had anovulatory infertility and 303 patients had normo-ovulatory infertility in a public university-affiliated in vitro fertilization center. Sperm mROS and hydrogen peroxide testing were performed by flow cytometry and analyzed for their relationship with embryo development indices on days 1–6 after IVF. Multivariate logistic regression analysis was used to control for female potential confounders. The nonlinear effects of sperm ROS on embryo development were analyzed by the Restricted cubic spline (RCS) method.

**Results:**

1. Multivariate linear logistic regression analysis showed that high proportion of mROS positive sperm improved the 2PN rate (OR = 1.325, 95% CI: 1.103–1.595), day 3 embryo utilization rate (OR = 1.362, 95% CI: 1.151–1.614) and good-quality day 3 embryo rate (OR = 1.391, 95% CI: 1.089–1.783) in patients with anovulatory infertility. High percentage of sperm mROS and hydrogen peroxide had adverse effects on cleavage-stage embryo and blastocyst development in patients with normo-ovulatory infertility. 2. For patients with polycystic ovarian syndrome (PCOS) anovulatory infertility, there were significant distinct effects on embryo development indices between sperm mROS and hydrogen peroxide, and the increased rate of sperm mROS improved the good-quality day 3 embryo rate (OR = 1.435, 95% CI: 1.045–1.981); however, high percentage of sperm hydrogen peroxide reduced the blastocyst utilization rate (OR = 0.555, 95% CI: 0.353–0.864) and the good-quality blastocyst rate (OR = 0.461, 95% CI: 0.292–0.718). 3. Multivariate RCS analysis revealed that sperm ROS had a nonlinear (such as a parabolic curve) effect on embryo development in patients with anovulatory infertility (*P* < 0.05), and either greatly increased or greatly decreased affected cleavage-stage embryo and blastocyst development. The effects of sperm ROS in patients with normo-ovulatory infertility were both linear and nonlinear.

**Conclusions:**

These findings indicate that contrary effects of sperm mROS on embryo development depending on whether patients treated with IVF cycles had normal ovulation. Regardless of whether the patients ovulated normally, increased sperm hydrogen peroxide rate damaged blastocyst development. It is necessary to evaluate male sperm ROS levels and the female ovulatory state to determine an individualized intervention plan before starting cycles, as this may be beneficial for infertile couples.

## Background

Reactive oxygen species (ROS) are oxygen radicals generated by aerobic metabolism of mitochondrial electron chains. Conventional knowledge indicates that physiological ROS production promotes embryo development and that excess ROS negatively impact embryo development. Previous studies have reported that the decreased sperm quality caused by elevated sperm ROS may lead to embryo arrested development or even apoptosis [1, 2]. A growing number of studies have reported that human mature oocytes have the ability to repair sperm ROS damage and alleviate the adverse effects of low-quality sperm on embryo development [3–5], but there exists an upper limit of repair capability.

Due to regulatory and ethical concerns, it is difficult to directly detect the oxidative stress state of human oocytes and embryos to reveal the effects of sperm ROS on embryo development. There are a limited number of studies on the effects of sperm ROS on in vitro embryos, and most of these studies did not differentiate female infertility factors [6–8], ignoring the importance of oocyte quality in embryo development and the large differences in oocyte quality in women with infertility of different etiologies [9].

How do sperm ROS affect embryo development? Do elevated sperm ROS levels only have a negative effect on embryo development? Furthermore, can different types of sperm ROS have distinct effects on embryo development? There has been no relevant research on these issues until now. This paper is a prospectively investigation designed to study the effects of different types of sperm ROS (including mitochondrial ROS (mROS) and hydrogen peroxide) on embryos at different developmental stages in women with different infertility etiologies undergoing IVF cycles to provide a basis for individualized treatments for infertile couples.

## Methods

### Study design and participants

This prospective cohort study was performed at a public university-affiliated reproductive medicine center between July 2020 and July 2021. The inclusion criteria for this investigation were as follows: 1. females younger than 40 years old; 2. use of conventional IVF; 3. more than 5 retrieved oocytes and more than 3 cleavage-stage embryos used for blastocyst culture; and 4. normal chromosomal karyotypes in all couples. The exclusion criteria were as follows: 1. insufficient quantity of selected sperm for mROS and hydrogen peroxide testing; 2. use of intracytoplasmic sperm injection fertilization (ICSI); 3. female patients with complicating medical diseases such as thyroid dysfunction, hypertension, and diabetes; or 4. significant indications of male semen infection. A total of 393 cycles were included. Relevant clinical data were collected, including the age of the husband and wife, type of infertility, etiology of infertility, serum basic sexual hormone levels, body mass index (BMI), anti-Müllerian hormone (AMH) level, antral follicle count, medication time and dosage of gonadotropin (Gn), total dosage of Gn, number of retrieved oocytes, fertilization rate, cleavage rate, and number of good-quality embryos.

This study was approved by the Ethics Review Committee of local institution (the IRB Number: Shenzhen People’s Hospital LL-KY-2021360).

### Controlled ovarian stimulation

Conventional methods were used for controlled ovarian stimulation (COH), monitoring follicles, ovum pick up (OPU), fertilization and embryo culture. The most frequently used protocols were the gonadotropin-releasing hormone antagonist (GnRH-ant) protocol, luteal phase gonadotropin-releasing hormone agonist (GnRH-a) protocol and follicular phase gonadotropin-releasing hormone agonist protocol. Taking the fixed GnRH-ant protocol as an example, on the second or third day of the cycle, COH was started by administering a daily dose of recombinant follicle-stimulating hormone (FSH) (Puregon; Merck Sharp& Dohme, New Jersey, America). Follicular growth was monitored using transvaginal ultrasound examination, and pituitary blockage was performed using a gonadotropin -releasing hormone antagonist (ganirelix; Merck Sharp & Dohme, New Jersey, America) starting on day 5 of gonadotropin administration. When three or more follicles attained a mean diameter of 17 mm, human chorionic gonadotropin (hCG) 6000–10,000 IU SC was administered to trigger the final follicular maturation, and ultrasound-guided transvaginal oocyte retrieval was performed 34–36 hours after hCG administration.

### Sperm preparation and evaluation of ROS levels

**Fig. 1:**
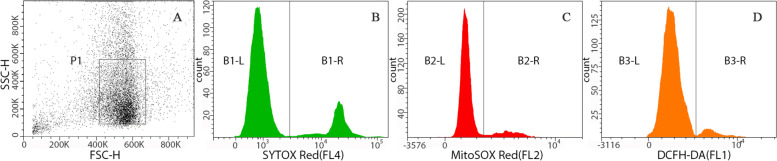
Flow cytometric out-gating of ROS sperm particles based on scatter properties and histogram after Triple-Staining. Sperm cells were selected using discontinuous density gradients method and stained with SYTOX Red, MitoSOX Red and DCFH-DA. **A** FSC/SSC dot plot obtained from a semen sample. A region (P1) is established to exclude debris. **B** SYTOX Red fluorescence histogram obtained within P1 region. The B1-L and B1-R peaks represent viable and nonviable sperm, respectively. **C** MitoSOX Red fluorescence distribution of viable sperm, were derived by gating on the B1-L population in panels B. The B2-R region represents ROS positive living sperm. **D** DCFH-DA fluorescence histogram obtained within B1-L region. The B3-R populations contains hydrogen peroxide positive live sperm events

Semen samples were collected and analyzed according to the World Health Organization guidelines. Semen selection and ROS analysis were performed on OPU day. Sperm samples were prepared using a two-layered density gradient centrifugation technique (40 and 80% isolate, Quinn’s ART-2040/2080). After preparation, a portion of the selected sperm were suspended in modified human tubal fluid (mHTF) (Quinn’s ART-1023) for sperm ROS analysis, and the other portion was kept for conventional fertilization in IVF cycles. Sperm mROS and hydrogen peroxide testing were performed by flow cytometry (a commercial kit provided by Shenzhen Bred Life Science Technology Inc. China). Sperm mROS were detected with a mitochondrial superoxide anion-specific fluorescent probe (MitoSOX Red), and sperm hydrogen peroxide was tested with a DCFH-DA probe. Dead spermatozoa were identified with a SYTOX Red probe, and the percentages of mROS and hydrogen peroxide in live sperm were analyzed. The testing methods were as follows: 0.5 ml of the selected sperm suspension and 5 μl of joint detection probe were added to a small glass tube (MitoSOX Red+DCFH-DA + SYTOX Red); at least 5000 sperm were analyzed by flow cytometry after an incubation of 15 min at 37 °C. The excitation wavelength of MitoSOX Red was between 510 and 580 nm (excitation, 488 nm; yellow fluorescence in the FL2 channel), that of DCFH-DA was between 500 and 530 nm (excitation, 488 nm; green fluorescence in the FL1 channel), and that of SYTOX Red was between 640 and 658 nm (excitation, 638 nm; red fluorescence in the FL4 channel) (Fig. 1). The percentages of mROS and hydrogen peroxide in the active sperm samples were analyzed and then reported with flow cytometry software.

### Embryo culture

Conventional IVF was performed. Mature oocytes observed on the first day after fertilization were calculated as the “effective counts of oocytes” to exclude the influence of immature oocytes on the data. Embryo quality evaluation was performed on days 1–3 and days 5–6 [10]. The IVF embryo development score was carried out by following the Vienna expert consensus [11].

### Statistical analysis

We used the following statistical tests. Basic clinical data, sperm quality parameters and indices of embryo development were analyzed separately and collectively for group comparison between the normo-ovulatory group and the anovulatory group. Descriptive statistics are presented as frequencies (percentages) or the mean ± SD. Associations with categorical variables were assessed using the χ2 test. Associations with quantitative and ordinal variables were assessed by logistic regression.

Associations with sperm ROS and indices of embryo development were analyzed by the nonparametric Spearman rank test.

When analyzing the effects of sperm ROS on embryo development in women with various infertility types and different normo-ovulatory/anovulatory types, the ROS indices were converted to the logarithmic before multivariate logistic regression. Supposing each oocyte has the same probability of developing into an embryo, a “successful event” was labeled as 1, and a “failure event” was labeled as 0. An odds ratio (OR) > 1 indicates a benefit to embryo development; an OR < 1 indicates a factor that is less favorable to embryo development.

The nonlinear relationship between sperm ROS and embryo development was assessed using Restricted cubic spline (RCS) analysis.

Maternal age, BMI, the medication time and dosage of Gn, the total dosage of Gn, AMH level, basal FSH level, COH protocol and other factors were treated as covariates in this study for multivariate logistic regression and multivariate RCS analysis.

All statistical analyses were performed using the software package SPSS 25.0 (IBM Corp. Armonk, NY, USA) and R 4.1.0. *P* < 0.05 was considered to be statistically significant.

## Results

### General conditions of the two groups

The baseline demographics of the patients in the anovulatory group and normo-ovulatory group are shown in Table 1. There was no significant difference in maternal or paternal age, BMI, any of the semen parameter or sperm ROS positive rate between the two groups(*P* > 0.05). There were no significant differences in embryo development indices between the two groups (*P* > 0.05).

**Table 1:** Comparison between the anovulatory group and the normo-ovulatory group

Factor	Ovulation disorders	Normo- ovulation	***P*** value	OR (95% CI)
No. of IVF cycles (*n*)	90	303		
Female age (y)	30.4 ± 3.7	31.2 ± 4.4	0.086^b^	1.051(0.993–1.112)
Female BMI (kg/m^2^)	22.6 ± 3.9	22.2 ± 3.0	0.388 ^b^	0.969(0.901–1.041)
Infertility				
Primary	52	139	0.047^a^	
Secondary	38	164		
Starting dose of Gn(U)	197.0 ± 48.0	227.2 ± 52.0	< 0.0001 ^b^	1.012(1.007–1.017)
Days of stimulation	10.1 ± 1.8	10.9 ± 1.9	< 0.0001 ^b^	1.292(1.120–1.490)
Total dose of Gn(U)	2036.6 ± 790.2	2602.1 ± 785.3	< 0.0001 ^b^	1.001(1.001–1.001)
b-FSH (mIU/ml)	7.1 ± 2.4	8.0 ± 2.1	0.002 ^b^	1.206(1.068–1.362)
b-LH (mIU/ml)	7.5 ± 4.5	4.6 ± 2.7	< 0.0001 ^b^	0.771(0.706–0.843)
b-FSH/bLH	1.3 ± 1.5	2.1 ± 1.4	< 0.0001 ^b^	3.019(2.021–4.511)
AFC (n)	20.2 ± 9.5	13.8 ± 6.6	< 0.0001 ^b^	0.894(0.861–0.929)
AMH (ng/ml)	7.4 ± 4.1	3.8 ± 2.2	< 0.0001 ^b^	0.653(0.588–0.727)
No. of oocytes retrieved	17.1 ± 7.1	13.8 ± 7.4	< 0.0001 ^b^	0.943(0.914–0.973)
Male age (y)	33.1 ± 5.3	33.9 ± 5.5	0.248 ^b^	1.207(0.982–1.075)
Sperm concentration (10^6^/ml)	94.2 ± 63.7	96.6 ± 66.3	0.760 ^b^	1.001(0.997–1.004)
Semen round cells (10^6^/ml)	0.59 ± 1.8	0.52 ± 0.7	0.739 ^b^	0.968(0.800–1.172)
Progressive motility (%)	58.6 ± 15.3	56.7 ± 15.1	0.306 ^b^	0.992(0.976–1.008)
Normal morphology (%)	7.8 ± 6.6	7.1 ± 3.9	0.216 ^b^	0.971(0.926–1.018)
Sperm mitochondrial ROS rate (%)	3.3 ± 2.4	3.4 ± 2.5	0.789 ^b^	1.013(0.921–1.114)
Sperm hydrogen peroxide rate (%)	0.5 ± 0.4	0.5 ± 0.5	0.420 ^b^	1.242(0.734–2.103)
Fertilization rate (%)	88.2 ± 15.9	89.0 ± 14.6	0.644 ^b^	1.433(0.311–6.597)
2PN rate (%)	68.9 ± 21.1	73.3 ± 18.4	0.055 ^b^	3.212(0.973–10.602)
≥3PN rate (%)	9.2 ± 10.7	9.2 ± 11.5	0.968 ^b^	0.958(0.120–8.657)
Cleavage rate (%)	96.6 ± 19.9	97.4 ± 18.9	0.669 ^b^	1.352(0.340–5.375)
Day 2 Embryo development rate (%)	57.7 ± 27.9	56.3 ± 25.7	0.655 ^b^	0.812(0.326–2.023)
Day 3 Embryo development rate (%)	38.8 ± 25.7	40.5 ± 26.5	0.605 ^b^	1.271(0.512–3.154)
Day 3 Embryo utilization rate (%)	58.3 ± 19.5	61.6 ± 20.5	0.179 ^b^	2.205(.697–6.980)
Good-quality day 3 embryo rate (%)	30.5 ± 20.5	33.7 ± 21.1	0.204 ^b^	2.115(0.666–6.717)
D5 Blastocyst utilization rate (%)	33.9 ± 26.7	30.7 ± 25.9	0.091 ^b^	0.223(0.089–0.557)
D5 Good-quality blastocysts rate (%)	29.6 ± 24.5	27.7 ± 24.0	0.105 ^b^	0.247(0.092–0.662)
D6 Blastocyst utilization rate (%)	28.2 ± 20.3	30.7 ± 27.8	0.473 ^b^	1.434(0.535–3.844)
D6 Good-quality blastocysts rate (%)	21.6 ± 18.1	22.4 ± 24.9	0.788 ^b^	1.161(0.391–3.449)
Blastocyst development rate (%)	66.8 ± 25.0	63.1 ± 32.0	0.352 ^b^	0.668(0.286–1.562)
Blastocyst utilization rate (%)	59.4 ± 23.4	53.4 ± 31.5	0.125 ^b^	0.508(0.214–1.206)
Good-quality blastocyst rate (%)	51.2 ± 24.2	46.2 ± 29.7	0.080 ^b^	0.374(0.154–0.911)

^a^ χ^2^ test

^b^ Associations with quantitative and ordinal variables assessed using logistic regression

### Linear correlation analysis (spearman) between sperm ros and in vitro embryo development indices

There was a weak linear positive correlation between sperm ROS and the development of cleavage-stage embryos (including the 4-cell rate on day 2 and the embryo utilization rate on day 3) in the anovulatory patients, and a weak linear negative correlation was found between sperm ROS and the polyspermy rate (*P* < .05). There was a weak linear negative correlation between sperm ROS and cleavage-stage embryo (day 3 embryo development rate) and blastocyst formation (day 5 good-quality blastocyst rate and day 6 blastocyst utilization rate) in normo-ovulatory infertile patients (*P* < .05) (Table 2).

**Table 2:** Linear correlation analysis (Spearman) between sperm ROS and embryo development indices

Factor	Ovulation disorders(***n*** = 90)				Normo-ovulation(***n*** = 303)			
	mROS		hydrogen peroxide		mROS		hydrogen peroxide	
	r	***P*** value	r	***P*** value	r	***P*** value	r	***P*** value
Fertilization rate (%)	0.068	0.523	0.016	0.881	−0.073	0.236	− 0.018	0.757
2PN rate (%)	0.164	0.122	0.097	0.364	−0.039	0.530	0.011	0.844
≥3PN rate (%)	−0.136	0.203	−0.211	0.046	0.026	0.672	0.004	0.951
Cleavage rate (%)	0.266	0.011	0.098	0.360	−0.035	0.576	−0.006	0.910
Day 2 Embryo development rate (%)	0.241	0.024	0.261	0.014	−0.045	0.466	−0.031	0.589
Day 3 Embryo development rate (%)	0.002	0.988	0.042	0.698	−0.057	0.361	−0.142	0.022
Day 3 Embryo utilization rate (%)	0.334	0.001	0.273	0.009	− 0.119	0.055	−0.025	0.551
Good-quality day 3 embryo rate (%)	0.182	0.086	−0.021	0.841	−0.117	0.059	0.034	0.349
D5 Blastocyst utilization rate (%)	0.113	0.328	0.071	0.538	−0.080	0.217	−0.057	0.216
D5 Good-quality blastocyst rate (%)	0.129	0.263	−0.087	0.451	−0.184	0.004	−0.075	0.293
D6 Blastocyst utilization rate (%)	0.139	0.228	−0.096	0.405	−0.192	0.003	−0.064	0.652
D6 Good-quality blastocyst rate (%)	−0.016	0.890	0.087	0.453	0.049	0.449	0.027	0.266
Blastocyst development rate (%)	−0.018	0.878	0.019	0.869	0.027	0.677	0.067	0.429
Blastocyst utilization rate (%)	0.173	0.133	0.079	0.495	−0.071	0.272	− 0.048	0.803
Good-quality blastocyst rate (%)	0.156	0.176	−0.035	0.764	−0.075	0.249	−0.015	0.666

### Effects of sperm ros on in vitro embryo development in various infertile factors

Because sperm mROS and hydrogen peroxide showed a biased distribution, the two indices were preprocessed by logarithm before multivariate logistic regression analysis.

Statistics showed that for anovulatory infertile patients, higher proportion of mROS positive sperm promoted embryo development based on the 2PN rate (OR = 1.325, 95% CI: 1.103–1.595, *P* < .01), day 3 embryo utilization rate (OR = 1.362, 95% CI: 1.151–1.614, *P* < .01) and good-quality day 3 embryo rate (OR = 1.391, 95% CI: 1.089–1.783, *P* < .01). However, for normo-ovulatory infertile patients, such as those with unexplained infertility, male infertility and tubal infertility, lower positive rate of sperm ROS (including mROS and hydrogen peroxide) were generally beneficial to cleavage-stage embryo and blastocyst formation (Table 3).

**Table 3:** Effects of sperm ROS on in vitro embryo development based on various infertility factors (multiple logistic regression analysis)

Infertility factors	n		2PN rate		Day 3 embryo development rate		Day 3 embryo utilization rate		Good-quality day 3 embryo rate		Good-quality blastocyst rate	
			OR (95%CI)	***P***	OR (95%CI)	***P***	OR (95%CI)	***P***	OR (95%CI)	***P***	OR (95%CI)	***P***
Ovulation disorder	90	log-mROS	1.325(1.103–1.595)	0.003	1.192(0.975–1.458)	0.087	1.362(1.151–1.614)	<.0001	1.391(1.089–1.783)	0.009	1.052(0.806–1.372)	0.711
		log-hydrogen peroxide	1.162(0.942–1.441)	0.159	1.048(0.840–1.309)	0.675	1.130(0.936–1.364)	0.204	0.902(0.691–1.177)	0.449	1.000(0.761–1.316)	0.999
Unexplained	75	log-mROS	0.920(0.715–1.180)	0.515	0.709(0.562–0.892)	0.003	0.685(0.559–0.836)	<.0001	0.746(0.567–0.978)	0.035	1.081(0.819–1.429)	0.582
		log- hydrogen peroxide	1.078(0.820–1.423)	0.592	0.827(0.631–1.082)	0.168	1.040(0.828–1.307)	0.735	1.116(0.826–1.511)	0.475	0.603(0.327–1.066)	0.891
Male	41	log-mROS	0.959(0.676–1.358)	0.816	0.830(0.559–1.230)	0.353	0.933(0.684–1.268)	0.658	0.628(0.362–1.068)	0.090	1.389(0.672–2.949)	0.381
		log- hydrogen peroxide	1.173(0.901–1.536)	0.242	0.729(0.535–0.984)	0.041	0.993(0.783–1.261)	0.954	1.013(0.665–1.544)	0.953	0.446(0.248–0.763)	0.005
Tubal	137	log-mROS	0.863(0.720–1.034)	0.112	1.000(0.821–1.217)	0.999	0.876(0.742–1.033)	0.116	0.867(0.674–1.112)	0.262	1.006(0.783–1.292)	0.963
		log- hydrogen peroxide	0.862(0.744–0.998)	0.047	0.916(0.783–1.071)	0.274	0.865(0.756–0.988)	0.033	1.009(0.823–1.235)	0.933	1.514(0.944–1.412)	0.163
Endometriosis	50	log-mROS	1.168(0.876–1.559)	0.291	1.114(0.824–1.511)	0.483	1.243(0.956–1.622)	0.106	0.812(0.555–1.181)	0.279	0.730(0.494–1.069)	0.109
		log- hydrogen peroxide	0.975(0.717–1.326)	0.871	0.894(0.650–1.228)	0.488	0.964(0.720–1.292)	0.808	1.106(0.744–1.649)	0.617	0.724(0.465–1.113)	0.146

### Effects of sperm ros on in vitro embryo development in patients with different types of ovulation disorders

Furthermore, 90 anovulatory patients were assigned to two groups: one group of patients with abnormal ovulation and PCOS and the other group comprising patients with abnormal ovulation without PCOS. In multiple logistic regression analysis, higher percentage of sperm mROS were beneficial to cleavage-stage embryo development, indicated by the 2PN rate (OR = 1.351, 95% CI: 1.257–1.931, *P* < .05), day 3 embryo utilization rate (OR = 1.985, 95% CI: 1.435–2.777, *P* < .01) and good-quality day 3 embryo rate (OR = 1.713, 95% CI: 1.106–2.707, *P* < .05), in patients with abnormal ovulation without PCOS. However, for those with abnormal ovulation and PCOS, contrary effects of sperm ROS on embryo development were observed. On the one hand, a higher rate of sperm mROS promoted the good-quality day 3 embryo rate (OR = 1.435, 95% CI: 1.045–1.981, *P* < .05), while on the other hand, high rate of sperm hydrogen peroxide appeared to be detrimental to embryo development, especially indicated by a reduction in the blastocyst utilization rate (OR = 0.555, 95% CI: 0.353–0.864, *P* < .01) and good-quality blastocyst rate (OR = 0.461, 95% CI: 0.292–0.718, *P* < .01) (Table 4).

**Table 4:** Effects of sperm ROS on in vitro embryo development in patients with different types of ovulation disorders (multiple logistic regression analysis)

Ovulation disorders Group	***n***		2PN rate		Day 3 embryo development rate		Day 3 embryo utilization rate		Good-quality day 3 embryo rate		Blastocyst utilization rate		Good-quality blastocyst rate	
			OR (95%CI)	***P***	OR (95%CI)	***P***	OR (95%CI)	***P***	OR (95%CI)	***P***	OR (95%CI)	***P***	OR (95%CI)	***P***
PCOS	50	log-mROS	0.959 (0.792–1.793)	0.101	1.379 (0.990–1.931)	0.059	1.044 (0.770–1.413)	0.782	1.435 (1.045–1.981)	0.026	1.026 (0.682–1.546)	0.902	1.125 (0.748–1.700)	0.572
		log-hydrogen peroxide	1.275 (0.815–2.003)	0.288	0.615 (0.434–0.864)	0.006	1.101 (0.783–1.547)	0.580	0.787 (0.557–1.106)	0.170	0.555 (0.353–0.864)	0.009	0.461 (0.292–0.718)	0.001
Abnormal ovulation without PCOS	40	log-mROS	1.351 (1.257–1.931)	0.042	1.192 (0.836–1.699)	0.331	1.985 (1.435–2.777)	<.0001	1.713 (1.106–2.707)	0.018	0.673 (0.402–1.105)	0.123	1.132 (0.707–.0825)	0.607
		log-hydrogen peroxide	0.558 (0.315–1.242)	0.072	1.263 (0.845–1.916)	0.261	1.526 (0.897–2.628)	0.122	1.408 (0.853–2.355)	0.184	1.500 (0.893–2.529)	0.124	0.883 (0.423–1.846)	0.740

### Nonlinear effects of sperm ros on embryo development after IVF cycles

**Fig. 2:**
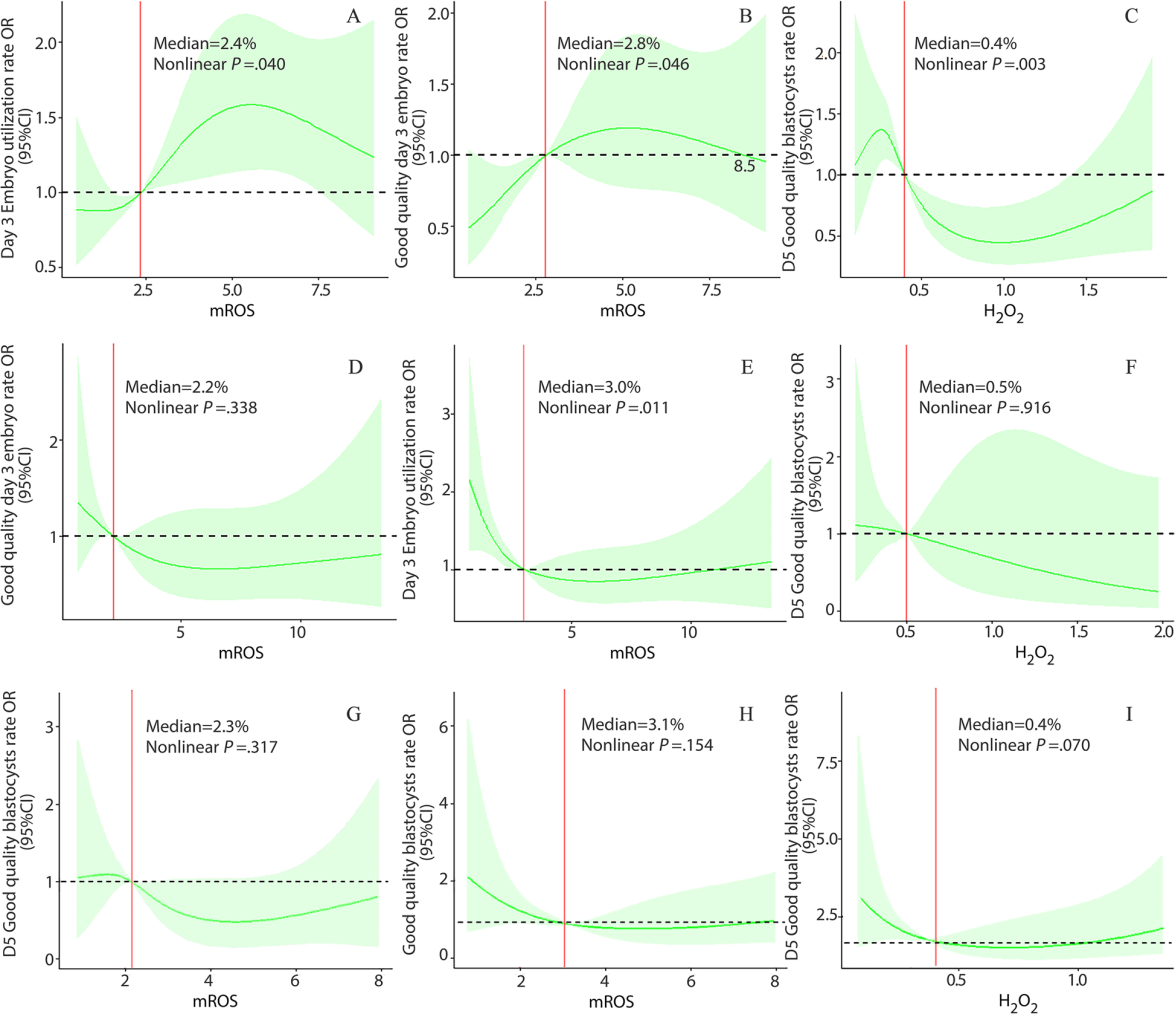
RCS analysis of the effect of sperm ROS on IVF embryo development in patients with anovulatory infertility (**A**-**C**) or normo-ovulatory infertility (**D**-**I**) (**D** and **E** for unexplained infertility and **F** for male sterility, G for tubal infertility, **H** and **I** for infertility due to endometriosis)

The nonlinear effect of sperm ROS on embryo development was observed through multivariate RCS analysis. Statistics showed that sperm ROS exert a nonlinear (such as a parabolic curve) effect on embryo development in patients with anovulatory infertility (*P* < 0.05). Among them, sperm mROS showed an n-type relationship with the day 3 embryo utilization rate and the good-quality day 3 embryo rate, and there was a J-type relationship between sperm hydrogen peroxide and the day 5 good-quality blastocyst rate (Fig. 2 A-C). The effects of sperm ROS on the embryo development of patients with normo-ovulatory infertility were slightly more complicated, and both linear and nonlinear forms existed simultaneously. However, the general trend was that a lower level of ROS was good for embryo development (Fig. 2 D-I).

## Discussion

The exact role of sperm ROS in early embryonic development has yet to be fully identified. Conventional evidence indicates two significant adverse impacts of sperm ROS. First, high levels of ROS directly impair sperm function. ROS are often generated in spermatozoa as a result of normal metabolic activity [12], and low levels of ROS are required for functions such as mobility, acrosome reaction and fertilization [13]. Spermatozoa are especially vulnerable to oxidative stress because of their limited antioxidative capacity. A series of oxidative stress reactions occur when sperm ROS exceed the upper limit of total antioxidative capacity, and those reactions can damage the sperm mitochondrial membrane, which will further increase ROS production and lead to mitochondrial dysfunction [14]. Second, the oxidative stress microenvironment of oocytes caused by sperm ROS transfer into oocytes during fertilization procedures and sperm DNA damage induced by ROS may have adverse effects on early embryo development, such as embryo growth retardation, stagnation or even apoptosis, eventually affecting clinical outcomes [15–17]. Much of the existing research agreed that increased ROS level was detrimental to embryo development, no matter the study subjects were human beings or animals.

However, previous clinical studies considered that sperm ROS levels cannot completely predict embryo viability and clinical outcome [9]. Possible reasons could include the following. 1. Different types of sperm ROS may have different effects on embryo development. 2. The calculation methods of embryo evaluation indices exhibit some irrationality. For example, the parameters of fertilization rate and embryo development are calculated with the number of retrieved oocytes as the denominator in standard practice, including immature and abnormal oocytes. The result is that adverse effects caused by oocytes are falsely attributed to sperm ROS, resulting in data deviations. 3. There are several confounding factors affecting embryo development and clinical outcomes, especially female factors [18]. 4. Significant differences in sperm ROS were observed due to the varying degrees of oocyte quality [12, 13].

Therefore, several strategies were adopted in this study. 1. The two indices of sperm mROS and hydrogen peroxide were detected simultaneously to observe the differences between different types of ROS on embryo development. 2. The “effective number of oocytes” was defined as mature oocytes observed after IVF routine fertilization for 17–19 h for calculation. The objective was to reduce the interference of some oocyte-derived factors on embryo development. 3. We used multivariate data analysis (covariates included female baseline demographics, endocrine state, COH protocol and dosage of Gn, etc.) to analyze the influence of sperm ROS on embryo development from linear and nonlinear perspectives. 4. Patients were divided and analyzed according to infertility etiology, and the effects of sperm ROS on embryo development in patients with normo-ovulatory and anovulatory infertility were comprehensively studied.

The three major findings of the current study are as follows: 1. low proportion of hydrogen peroxide positive sperm are conducive to blastocyst formation; 2. Sperm mROS affects mainly cleavage-stage embryo development; 3. The influences of sperm mROS on cleavage-stage embryos show very interesting opposing effects depending on whether the female patient has normal ovulation and an appropriately high percentage of sperm mROS, promoting embryo development in patients with anovulatory infertility but having negative effects in those with normo-ovulatory infertility. The findings that high rate of sperm mROS promote cleavage-stage embryo development in patients with anovulatory infertility has not been previously reported in the literature. The generation of this kind of phenomenon should be inseparable from oocyte quality in patients with anovulatory infertility.

The cause of ovulatory dysfunction is complex. At present, the literature on the relationship between human ROS and embryo development in female anovulatory patients has focused on PCOS. The points of research were concentrated to observation of phenomena between embryo development and ROS originating from follicular fluid [19], embryo culture media [20, 21], granulosa cells [22–24] and sperm [25].

Many studies have found that the oocyte utilization rate of PCOS patients is low after IVF cycles. Oxidative stress exists in follicular fluid and influences follicular growth and development. Oxidative damage interferes with the normal metabolic function of oocytes and granulosa cells and reduces the support and protection of granulosa cells to oocytes [26, 27]. Inflammation and mitochondrial dysfunction can increase the accumulation of ROS in granulosa cells [22, 23], which could affect oocyte development and fertilization, even embryo implantation [24]. These studies indicate that oocytes in PCOS infertility have been in a state of high activation of oxygen metabolism. However, according to the data of this study on the relationship between sperm mROS and cleavage-stage embryo, we speculate that the oxidative stress state of the follicular fluid microenvironment and granulosa cells may not be equal to the real situation of oxidative stress in oocytes and embryos. However, due to regulatory and ethical concerns, it is difficult to obtain human oocytes for scientific research. Furthermore, there is a lack of direct evidence to test mitochondrial ROS metabolism in oocytes surrounded by granulosa cells in PCOS infertility. The effect on and mechanism of different types of sperm ROS transfer to the oocyte during fertilization and subsequent embryo development remain unclear. In addition, from the perspective of sperm, our research data showed that the correlation between selected sperm mROS and hydrogen peroxide was weak (r = 0.125, *P* < 0.01). these results indicated that although sperm mROS and hydrogen peroxide are both ROS substances, their influences on embryo development may not be synchronized.

One study showed that mitochondrial dysfunction existed in germinal vesicle (GV) and metaphase II oocytes of a insulin-resistant PCOS mouse, and MII oocytes displayed a decrease in the ATP content and the mitochondrial DNA (mtDNA) copy number and an increase in the hydrogen peroxide level [28]. Another research reported that mitochondrial energy deficiency in mouse oocytes resulted in a decreased oocyte nuclear maturation rate and ROS generation, but did not affect the fertilization rate [29].

Why does an appropriate high-rate sperm mROS promote cleavage-stage embryo development in patients with ovulation disorders? We speculate that this is due to the low level of mitochondrial reactive oxygen species in the mature oocytes of patients with ovulation disorder. Mitochondrial dysfunction may exist in the oocytes of patients with infertility due to ovulation disorders, especially in PCOS patients, and ATP in short supply may lead to insufficient mitochondrial ROS production. Indicated by the animal experiments, sperm mitochondria are distributed among the blastomeres after fertilization, gradually decrease with embryo proliferation, and sperm mitochondria are undetectable at the blastocyst stage [30, 31]. The suitable concentration of mitochondrial ROS is an important signaling messenger and has been shown to promote the proliferation and differentiation of the embryo. Reducing the mitochondrial ROS level of the pig embryo will weaken embryo mitochondrial biogenesis [32]. We hypothesized that mROS carried by sperm are distributed among the blastomeres along with sperm mitochondria after fusing with oocytes, which may just fill the gap of oocyte mROS. However, low level or excess elevation of sperm mROS transferring into the oocyte may result in insufficient or excessive embryo mROS, which is not conducive to cleavage-stage embryo growth. Therefore, the deductions need to be further confirmed.

Previous researchers used the luminol-peroxidase chemiluminescence method to test human sperm hydrogen peroxide and observed that hydrogen peroxide had a negative effect on blastocyst development [24]. Another study found that the hydrogen peroxide level in day 3 culture medium was negatively correlated with IVF/ICSI blastocyst formation [20]. These conclusions are basically consistent with the result of our study. However, the sensitivity of the luminol peroxidase detection system for the detection of extracellular reactive oxygen species in non-leukocyte pollution, being less than that of flow cytometry, is insufficient [33].

Multiple logistic regression analysis indicated that sperm ROS had a significant effect on early embryo development in the current study, but the OR value was not very high. This is due to the complexity of factors affecting embryo development; on the other hand, the logistic regression analysis model was used only for linear data analysis. We further revealed the complicated effects of sperm ROS on embryo development through multifactor RCS analysis. First, sperm mROS and hydrogen peroxide presented a nonlinearity in embryonic development in patients with ovulation disorders; that is, sperm ROS promoted or inhibited embryo development only at an appropriate level, and sperm ROS at excessively high or low positive rate were disadvantageous to embryonic growth. The effect of mROS on cleavage-stage embryos was positive, and that of hydrogen peroxide on blastocyst formation was negative (Fig. 2 A-C). Second, the effects of sperm ROS showed two mixed modes, both linear and nonlinear, but the linear effect existed mainly in patients with normo-ovulatory infertility. Generally, sperm ROS has a negative effect (Fig. 2 D-I); specifically, a high proportion of ROS positive sperm is not conducive to embryo growth, which is consistent with most research results [15]. The RCS analysis showed that the cleavage-stage embryo developed much better when the mROS content of male selected sperm was in range of 2.4–8.5% in patients with ovulation disorders, overall. A sperm mROS positive rate less than 2.2% was good for embryo growth in patients with normo-ovulatory infertility; the hydrogen peroxide positive rate below 0.4% in selected sperm was beneficial to blastocyst formation regardless of whether the patient had an ovulation disorder.

There are also some limitations in this research. For example, the findings remain to be confirmed further from a clinical point of view. Additionally, the methods used for sperm ROS assays in this study lack quantitative analysis of ROS.

## Conclusions

Overall, this study found that sperm ROS had significant effects on embryo growth and development after IVF cycles. These results seem to indicate that different types of sperm ROS have different effects on embryo growth and development, increase the hydrogen peroxide positive rate in selected sperm affected blastocyst development, and influence sperm mROS in cleavage-stage embryos, with opposing effects depending on whether the female patient has normal ovulation. For those with anovulation, an appropriate high-positive rate sperm mROS promoted embryo growth, and increased sperm mROS inhibited embryo development in normo-ovulation patients, and their mechanisms need to be further studied. We believe that it is necessary to assess the male sperm ROS level and female ovulatory state before starting IVF cycles. An individualized intervention for males, including drug therapy or adjusting the proportional of oxidizers and antioxidants in culture medium, is expected to improve the oocyte utilization rate and embryo quality in infertile couples after IVF cycles.

## Data Availability

The datasets used are analyzed are during the current study are available from the corresponding author on reasonable request.

## Abbreviations

ROS: reactive oxygen species
mROS: mitochondrial ROS
IVF: in vitro fertilization
RCS: restricted cubic spline
PCOS: polycystic ovarian syndrome
BMI: body mass index
AMH: anti-Müllerian hormone
Gn: gonadotropin
OPU: ovum pick up
GnRH-ant: gonadotropin-releasing hormone antagonist
GnRH-a: gonadotropin-releasing hormone agonist
FSH: follicle-stimulating hormone
hCG: human chorionic gonadotropin
mHTF: modified human tubal fluid
MitoSOX Red probe: mitochondrial superoxide anion-specific fluorescent probe
GV: germinal vesicle
mtDNA: mitochondrial DNA
